# Joint Design of Polar Coding and Physical Network Coding for Two−User Downlink Non−Orthogonal Multiple Access

**DOI:** 10.3390/e25020233

**Published:** 2023-01-27

**Authors:** Zhaopeng Xie, Pingping Chen, Yong Li

**Affiliations:** 1 College of Physics and Information Engineering, Fuzhou University, Fuzhou 350108, China; 2 College of Computer Science, Chongqing University, Chongqing 400044, China

**Keywords:** polar code, two-user downlink non-orthogonal multiple access channels, physical layer network coding

## Abstract

In this paper, we propose a joint polar coding and physical network coding (PNC) for two−user downlink non−orthogonal multiple access (PN−DNOMA) channels, since the successive–interference–cancellation–aided polar decoding cannot be optimal for finite blocklength transmissions. In the proposed scheme, we first constructed the XORed message of two user messages. Then, the XORed message was superimposed with the message of the weak User 2 for broadcast. By doing so, we can utilize the PNC mapping rule and polar decoding to directly recover the message of User 1, while at User 2, we equivalently constructed a long−length polar decoder to obtain its user message. The channel polarization and decoding performance can be greatly improved for both users. Moreover, we optimized the power allocation of the two users with their channel conditions by considering the user fairness and the performance. The simulation results showed that the proposed PN−DNOMA can achieve performance gains of about 0.4−0.7 dB over the conventional schemes in two−user downlink NOMA systems.

## 1. Introduction

Non-orthogonal multiple access (NOMA) is a promising multiple access technique in 5G communications to increase system throughput and accommodate massive connectivity [1]. Compared to traditional orthogonal multiple access (OMA), NOMA allows multiple users to simultaneously transmit signals using the same time/frequency radio resources, but different power levels [2]. The key advantage of NOMA is exploring the extra power domain to further increase the number of supportable users. Specifically, users are identified by their channel conditions. Those with good channel conditions are called strong users, and the others are called weak users [3]. For the sake of fairness, less power is allocated to strong users at the transmitter side. In this way, the transmitter sends the superposition of signals with different power levels, and the receiver applies successive interference cancellation (SIC) to strong users to realize multi-user detection. Consider a typical NOMA system, which consists of a base station (BS) and two user nodes. Ref. [4] proposed power allocation (PA) algorithms to maximize the ergodic capacity under the power and rate constraints of the weak user. With fixed power coefficients, Ref. [5] considered the exact bit error rate (BER) analysis of a two-user NOMA system using square quadrature amplitude modulation (QAM). Ref. [6] proposed a joint adaptive *M*-QAM modulation and power adaptation. While the BS is equipped with multiple antennas, Ref. [7] proposed a matched-filter (MF) preceding algorithm and analyzed the required power consumption under SINR constraints for both users, as well as the power-outage tradeoff. Ref. [8] analyzed the quasi-degradation probability and proposed an analytical framework to characterize the optimality of NOMA over Rician fading channels. Moreover, Reference [9] investigated the achievable rate region in the large-system limit of regular sparse NOMA with two users. Given a minimum target rate for the individual users, Reference [10] analyzed the outage probability with respect to the total data rates.

In conventional NOMA systems, the SIC is employed for signal decoding, which is optimal for sufficient long blocklength transmissions [11]. However, for finite blocklength transmissions, the joint decoding instead of SIC has been shown to be better [12]. By using the random channel coding theorem, Reference [13] proposed a NOMA implementation without SIC and proved that the conditional achievable sum rate given channel gains can be achieved by this scheme. Polar code, proposed by Arikan in 2006, can achieve the symmetric channel capacity of binary discrete memoryless channels under a successive cancellation (SC) or a cyclic−redundancy−check−aided SC−list (CA−SCL) decoder, as the code length goes to infinity [14]. Considering the decoding error probability and PA optimization, a joint polar decoding and SIC decoding strategy (PC−SIC) was proposed [12]. PC-SIC aims to maximize the effective throughput at the central user under the minimum required effective throughput constraint at the cell edge user. However, this scheme mainly considers the individual decoding by users, and the superposition of signals is performed to ensure that the inter-user interference can be successfully removed by SIC at the receiver.

Moreover, the physical-layer network coding (PNC) can significantly enhance the spectrum efficiency and the network throughput by utilizing network interference [15,16]. The concept of PNC lies in that the relay maps the overlapped received signals from two users to a network coding (NC) message. The channel coding and efficient decoding were developed for polar-coded PNC [17]. Network-coded multiple access (NCMA) was proposed for NOMA systems [18], which estimates an NC message and then a single-user message.

In this paper, by exploiting the principles of the polar code and PNC, we propose a joint polar coding and PNC over two-user downlink NOMA (PN−DNOMA) systems, which can convert the inter-user interference into a useful message. In particular, User 1 can use the PNC mapping rule for direct decoding, while User 2 can construct a longer-length decoder from the received signal, and the channel polarization effect can be greatly improved. In the proposed scheme, the BS transmits the superimposed message of the XORed message of two users and the message of the weak User 2. This is different from the conventional downlink NOMA schemes, where the superimposed message of two users is broadcast [19]. Then, we can directly recover the message of User 1 aided by PNC mapping from the received signal, and the message of User 2 is estimated by using a constructed long−length polar decoder. In addition, we determined the optimal power allocation of the two users given the channel condition to improve the user fairness and performance. The simulation results showed that the proposed PN−DNOMA can achieve performance gains of about 0.4–0.7 dB over the conventional PC−SIC in the two-user downlink NOMA system.

The rest of this paper is organized as follows. Section 2 briefly reviews the system model and polar code. Section 3 explicitly gives the proposed PN-DNOMA. Section 4 presents the simulation results, and Section 5 concludes the paper.

## 2. Background and System Model

### 2.1. System Model

**Figure 1:**
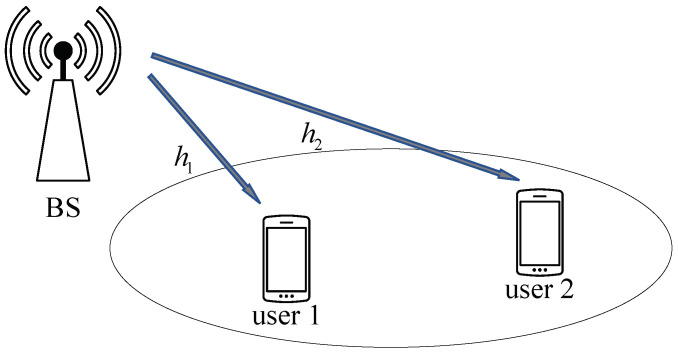
Block diagram of a two-user downlink NOMA system.

We considered a downlink NOMA system with two user nodes and a BS, as shown in Figure 1. The channel gain from the BS to user *s* is expressed as $h_{s}$, s∈{1,2}. In particular, $h_{s}=d_{s}^{-\varphi }g_{s}$, with $d_{s}$ being the distance from the BS to the user *s*, where φ is the loss exponent and $g_{s}∼CN\left(0,1\right)$ [20]. Without loss of generality, we assumed that User 1 and User 2 are the strong user and weak user from the BS, respectively. In this paper, for convenience, we use the notation $a_{i}^{j}$ to denote a sequence $\left(a_{i},a_{i+1},\cdots ,a_{j}\right)$ and $a_{i,v}^{j}$ to denote the *v*-th message in the superimposed signal, v∈{1,2}. Let $u_{1,s}^{Z}$ denote the source bits of length *Z* from the BS sent to user *s*, s∈{1,2}. After polar encoding for $u_{1,s}^{Z}$, we can have the length−*Z* codeword ${\bar{x}}_{1,s}^{Z}$ of user *s*. In the conventional downlink NOMA [21], the two-user codewords are modulated and superimposed at the BS, then broadcast to the two users.

In this paper, at the BS, we first obtain the XORed message $x_{1,1}^{Z}={\bar{x}}_{1,1}^{Z}⊕{\bar{x}}_{1,2}^{Z}$. For notational simplicity, we denote the message of User 2 by $x_{1,2}^{Z}={\bar{x}}_{1,2}^{Z}$. Afterward, $x_{1,v}^{Z}$ is the BPSK modulated to the signal $r_{1,v}^{Z}$, v=1,2. Then, we can construct the superimposed signal of the XORed $r_{1,1}^{Z}$ and the message $r_{1,2}^{Z}$ for broadcast. The received signal at the user *s* can be given by

(1) $$y_{s}=h_{s}\sum_{v=1}^{2}\sqrt{p_{v}}r_{1,v}^{Z}+w_{1,s}^{Z},$$

where $P_{v}$ denotes the transmit power for $r_{v}$, subjected to the total transmit power $P_{t}$, i.e., $P_{1}+P_{2}=P_{t}$ and $P_{2}>P_{1}$. *w* denotes a Gaussian random noise with variance $\sigma^{2}$, between user *s* and the BS. $h_{s}$ are assumed to be known at the BS, which may be obtained by a feedback channel from the users [22].

### 2.2. Channel Polarization

Let W:X→Y denote a binary-input discrete memoryless channel (B-DMC) with the input alphabet X={0,1}, output alphabet Y, and channel transition probabilities {W(y|x):x∈X,y∈Y}. For a length-*N* polar code, the source block $u_{1}^{N}$ consists of information bits $u_{\Lambda }\in {\left\{0,1\right\}}^{K}$ and frozen bits $u_{\Lambda^{c}}\in {\left\{0\right\}}^{N-K}$, where Λ denotes information bits and $\Lambda^{c}$ denotes frozen bits. In this paper, we adopted a systematic polar code [23], and the code bits $x_{1}^{N}=\left(x_{1},x_{2},\ldots x_{N}\right)$, $x_{1}^{N}\in {\left\{0,1\right\}}^{N}$, given by

(2) $$x_{1}^{N}=u_{1}^{N}G_{N},$$

where $G_{N}$ is the *N*-dimensional generator matrix and $G_{N}=F^{⊗n}$, where ⊗ denotes the Kronecker product $F=\left[\begin{array}{cc} 1 & 0\\ 1 & 1 \end{array}\right]$, $n=\log_{2}\left(N\right)$. The channels are merged and split into bit-channels $W_{N}^{\left(i\right)}$, i=1,2,…,N. Let $W_{N}^{\left(i\right)}\left(y_{1}^{N},{\hat{u}}_{1}^{i-1}|u_{i}\right)$ denote the *i*−th channel transition probability with input bit $u_{i}$ and outputs $y_{1}^{N},u_{1}^{i-1}$, given by

(3) $$W_{N}^{\left(i\right)}\left(y_{1}^{N},u_{1}^{i-1}|u_{i}\right)≜\sum_{u_{i+1}^{N}\in X^{N-i}}\frac{1}{2^{N-1}}W_{N}\left(y_{1}^{N}|u_{1}^{N}\right).$$

The *K* most-reliable split bit−channels are determined to transmit the information bits, and the rest are used as frozen bits, i.e., zeros. The decoder calculates the LLR of the *i*-th bit $u_{i}$ as

(4) $$L_{N}^{\left(i\right)}≜\ln\left(\frac{W_{N}^{\left(i\right)}\left(y,\hat{u}{\;}_{1}^{i-1}|0\right)}{W_{N}^{\left(i\right)}\left(y,\hat{u}{\;}_{1}^{i-1}|1\right)}\right),\;\;i=1,2,\cdots ,N,$$

where ${\hat{u}}_{1}^{i-1}$ is the estimation of the vector $u_{1}^{i-1}$. During the decoding, the LLRs for odd channels and for even channels can be, respectively, computed via recursion:

(5) $$\begin{array}{cc} \; & L_{N}^{\left(2i-1\right)}\left(y_{1}^{N},\hat{u}{\;}_{1}^{2i-2}\right)=L_{N/2}^{\left(i\right)}\left(y_{1}^{N/2},\hat{u}{\;}_{1,o}^{2i-2}⊕\hat{u}{\;}_{1,e}^{2i-2}\right)\\ \; & ⊞L_{N/2}^{\left(i\right)}\left(y_{N/2+1}^{N},\hat{u}{\;}_{1,e}^{2i-2}\right), \end{array}$$

and

(6) $$\begin{array}{cc} \; & L_{N}^{\left(2i\right)}\left(y_{1}^{N},\hat{u}{\;}_{1}^{2i-1}\right)={\left(-1\right)}^{{\hat{u}\;}_{2i-1}}L_{N/2}^{\left(i\right)}\left(y_{1}^{N/2},\hat{u}{\;}_{1,o}^{2i-2}⊕\hat{u}{\;}_{1,e}^{2i-2}\right)\\ \; & +L_{N/2}^{\left(i\right)}\left(y_{N/2+1}^{N},\hat{u}{\;}_{1,e}^{2i-2}\right), \end{array}$$

where ${\hat{u}}_{1,o}^{2i-2}$ and ${\hat{u}}_{1,e}^{2i-2}$ are the subvectors of estimated bits with odd and even indices, respectively. The box−plus operation ⊞ is defined as $L_{1}⊞L_{2}=\log\left(\left(1+e^{L_{1}+L_{2}}\right)/\left(e^{L_{1}}+e^{L_{2}}\right)\right)$ [24].

## 3. Polar Coded for NOMA

### 3.1. Proposed PN-DNOMA Scheme

Recall that the encoding matrix can be obtained by the *n*−times Kronecker product of the 2×2 polarization kernel *F* and operated in the binary field [14]. Thus, for a length−N polar coding, the source block $u_{1}^{N}$ to be encoded into $x_{1}^{N}$ equivalently consists of two steps. In the first step, the *M* sub−block $u_{1}^{N}=\left(u_{1}^{Z},u_{Z+1}^{2Z},\cdots ,u_{(M-1)Z+1}^{N}\right)$ is encoded by $F^{⊗n-m}$ to generate the sub−codewords $\left({\bar{x}}_{1}^{Z},{\bar{x}}_{Z+1}^{2Z},\cdots ,{\bar{x}}_{(M-1)Z+1}^{N}\right)$, respectively, Z=N/M and $m=\log_{2}\left(M\right)$. In the second step, the resulting sub−codewords are encoded by ${\bar{G}}_{M}=F^{⊗m}$ into $x_{1}^{N}$. This encoding process is given by

(7) $$\begin{array}{c} x_{1}^{N}=u_{1}^{N}G_{N}\;\\ \;\;\;=u_{1}^{N}\left(F^{⊗n-m}⊗F^{⊗m}\right)\;\\ \;\;\;=u_{1}^{N}\left[\begin{array}{ccc} F^{⊗n-m} & \cdots & 0\\ \vdots & \ddots & \vdots \\ F^{⊗n-m} & \cdots & F^{⊗n-m} \end{array}\right]\;\\ \;\;\;=({\left(u_{1}^{Z}F^{⊗n-m}\right)}^{T},{\left(u_{Z+1}^{2Z}F^{⊗n-m}\right)}^{T},\cdots ,\;\\ \;\;\;{\left(u_{(M-1)Z+1}^{N}F^{⊗n-m}\right)}^{T}){\bar{G}}_{M}\;\\ \;\;\;=\left({\left({\bar{x}}_{1}^{Z}\right)}^{T},{\left({\bar{x}}_{Z+1}^{2Z}\right)}^{T},\cdots ,{\left({\bar{x}}_{(M-1)Z+1}^{N}\right)}^{T}\right){\bar{G}}_{M}. \end{array}$$

In the two−user NOMA, from (7), we assumed that the desired message for the user *s* is ${\bar{u}}_{1,s}^{Z}=\left({\bar{u}}_{1,s},{\bar{u}}_{2,s},\ldots ,{\bar{u}}_{Z,s}\right)$, which is a sub-block of the source block $u_{1}^{N}=\left(u_{1},u_{2},\cdots ,u_{N}\right)$, i.e., $u_{(s-1)Z+1}^{sZ}={\bar{u}}_{1,s}^{Z}$, N=MZ, and M=2.

**Figure 2:**
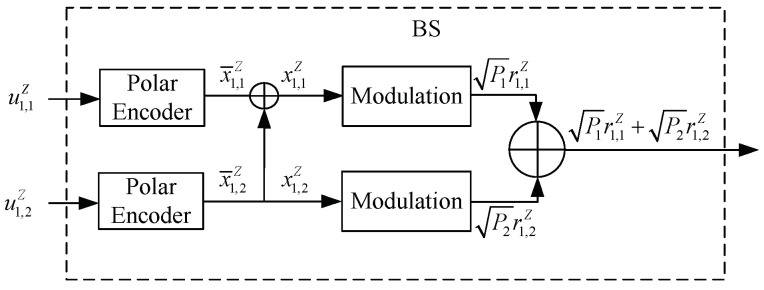
Block diagram of the transmitter in two-user PN−DNOMA.

In particular, from the encoding principles in (7), the individual polar coding of length-*Z* for two users at the BS is equivalently transformed to a single polar coding of a larger length *N*, N=2Z, as shown in Figure 2. Thus, at the BS, we can have the sub-codeword $x_{1,1}^{Z}$ as

(8) $$\begin{array}{c} x_{1,1}^{Z}={\bar{x}}_{1,1}^{Z}⊕{\bar{x}}_{1,2}^{Z}\;\\ \;\;\;\;\;\;\;={\bar{u}}_{1,1}^{Z}F^{⊗n-m}⊕{\bar{u}}_{1,2}^{Z}F^{⊗n-m}, \end{array}$$

which is also the XORed signal of the two user codewords. Similarly, we can generate the sub−codeword of $x_{1,2}^{Z}$ as

(9) $$x_{1,2}^{Z}={\bar{x}}_{1,2}^{Z}={\bar{u}}_{1,2}^{Z}F^{⊗n-m}.$$

Then, the $x_{1,s}^{Z}$ is BPSK−modulated to $r_{1,s}^{Z}$, s=1,2, which are then superimposed and sent to the users. In user node *s*, the *i*−th received signal $y_{i,s}$ is given by

(10) $$y_{i,s}=h_{s}\left(\sqrt{P_{1}}r_{i,1}+\sqrt{P_{2}}r_{i,2}\right)+w_{i,s}.$$

We define a set that collects four possible superimposed signals at user *s* as $S=\{h_{s}\left(\sqrt{P_{1}}+\sqrt{P_{2}}\right),h_{s}\left(\sqrt{P_{1}}-\sqrt{P_{2}}\right),h_{s}\left(-\sqrt{P_{1}}+\sqrt{P_{2}}\right),-h_{s}\left(\sqrt{P_{1}}+\sqrt{P_{2}}\right)\}$, where the *j*−th element $s_{j}$ in S has the a priori probability $\mathrm{Pr}(s_{j})=1/4$, j=0,1,2,3. At the user *s*, the probability of the received $y_{i,s}$ given the transmit signal $s_{j}$ is

(11) $$\mathrm{Pr}\left(y_{i,s}|s_{j}\right)=\frac{1}{\beta \sqrt{2\pi }\sigma }\exp\left(\frac{{\left(y_{i,s}-s_{j}\right)}^{2}}{2\sigma^{2}}\right),$$

where β is a normalization factor that ensures $\sum_{j=0}^{3}\mathrm{Pr}(y_{i,s}|s_{j})=1$. Moreover, we define another XOR-ed message $x_{1,o}^{Z}=x_{1,1}^{Z}⊕x_{1,2}^{Z}$ for the superimposed signals, $x_{1,o}^{Z}=\left(x_{1,1}⊕x_{1,2},x_{2,1}⊕x_{2,2},\ldots ,x_{Z,1}⊕x_{Z,2}\right)$. Table 1 summarizes the PNC mapping rules for the coded bit and the received signal.

At the user *s*, we use ${\bar{L}}_{i,s,1}$, ${\bar{L}}_{i,s,2}$ and ${\bar{L}}_{i,s,xor}$ to denote the initial LLRs of the *i*-th bit in $x_{1,1}^{Z}$, $x_{1,2}^{Z}$ and $x_{1,o}^{Z}$, respectively, which can be computed by

(12) $$\left\{\begin{array}{c} {\bar{L}}_{i,s,1}=\ln\left(\frac{\mathrm{Pr}\left(y_{i,s}|s_{0}\right)+\mathrm{Pr}\left(y_{i,s}|s_{2}\right)}{\mathrm{Pr}\left(y_{i,s}|s_{1}\right)+\mathrm{Pr}\left(y_{i,s}|s_{3}\right)}\right),\;\\ {\bar{L}}_{i,s,2}=\ln\left(\frac{\mathrm{Pr}\left(y_{i,s}|s_{0}\right)+\mathrm{Pr}\left(y_{i,s}|s_{1}\right)}{\mathrm{Pr}\left(y_{i,s}|s_{2}\right)+\mathrm{Pr}\left(y_{i,s}|s_{3}\right)}\right),\;\\ {\bar{L}}_{i,s,xor}=\ln\left(\frac{\mathrm{Pr}\left(y_{i,s}|s_{0}\right)+\mathrm{Pr}\left(y_{i,s}|s_{3}\right)}{\mathrm{Pr}\left(y_{i,s}|s_{1}\right)+\mathrm{Pr}\left(y_{i,s}|s_{2}\right)}\right). \end{array}\right.$$

At User 1, from (8) and (9), we can see that the message ${\bar{x}}_{1,1}^{Z}$ can be derived from the XORed message $x_{1,o}^{Z}$: i.e.,

(13) $$\begin{array}{c} {\bar{x}}_{1,1}^{Z}=x_{1,o}^{Z}\;\\ \;\;\;\;\;\;\;=x_{1,1}^{Z}⊕x_{1,2}^{Z}\;\\ \;\;\;\;\;\;\;=\left({\bar{x}}_{1,1}^{Z}⊕{\bar{x}}_{1,2}^{Z}\right)⊕{\bar{x}}_{1,2}^{Z}. \end{array}$$

Then, the *i*-th initial LLR values for ${\bar{x}}_{i,1}$ can be given by

(14) $$L_{i,1}={\bar{L}}_{i,1,xor},\;\;\;\;\;i\in \left\{1,2,\cdots ,Z\right\}.$$

**Figure 3:**
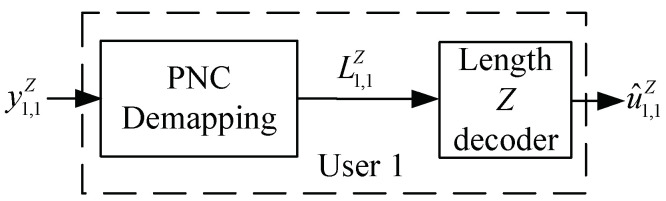
Block diagram of the receiver at User 1 in PN−DNOMA.

Afterward, the LLR vector $L_{1,1}^{Z}=\left(L_{1,1},L_{2,1},\ldots ,L_{Z,1}\right)$ is fed into the polar decoder with codelength *Z* to estimate the source bits ${\hat{u}}_{1,1}^{Z}$, as shown in Figure 3.

**Figure 4:**
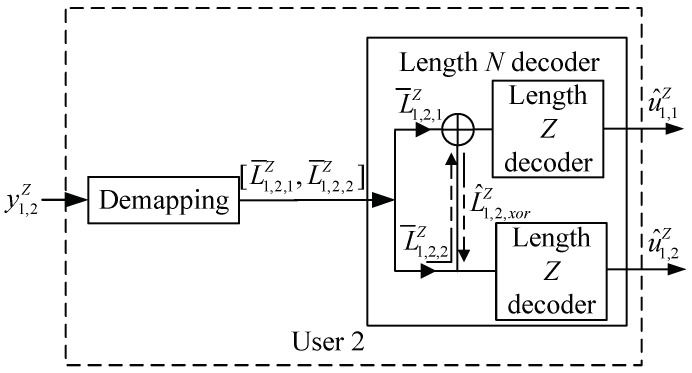
Block diagram of the receiver at User 2 in PN−DNOMA.

From (7), a polar code with codelength N=2Z is constructed for User 2. As shown in Figure 4, $L_{1,2}^{N}$ denotes the initial LLR vector of this code, $L_{1,2}^{N}=\left(L_{1,2},L_{2,2},\ldots ,L_{N,2}\right)$, and the *i*-th initial value is given by

(15) $$L_{i,2}=\left\{\begin{array}{c} {\bar{L}}_{i,2,1},\;\;\;\;\;\;\;\;\;i\in \left\{1,2,\cdots ,Z\right\},\;\\ {\bar{L}}_{i-Z,2,2},\;\;\;\;\;i\in \left\{Z+1,Z+2,\ldots ,N\right\}. \end{array}\right.$$

where ${\bar{L}}_{i,2,1}$ and ${\bar{L}}_{i,2,2}$ denote the initial LLR values of $x_{i,1}$ and $x_{i,2}$, respectively.

In this case, we can recover the source bits $\left\{{\hat{u}}_{1,1}^{Z},{\hat{u}}_{1,2}^{Z}\right\}$ at User 2 in the polar decoder with length *N*. We can decode ${\hat{u}}_{1,1}^{Z}$ with the initial LLR $L_{1,2}^{N}$ in the polar decoder. In particular, we can recover the source bits ${\hat{u}}_{1,2}^{Z}$ in two ways. First, the source bit ${\hat{u}}_{1,2}^{Z}$ can be directly decoded by using $L_{Z+1,2}^{N}$ in the length-*Z* polar decoder. Second, by the characteristics of polar decoding, let ${\hat{L}}_{1,2}^{Z}$ denote the LLR value of the XORed message of $L_{1,2}^{Z}$ and ${\hat{u}}_{1,1}^{Z}F^{n-1}$ [25]. We can see that ${\hat{L}}_{1,2}^{Z}$ can also be used to decode ${\bar{x}}_{1,2}^{Z}$. Thus, $L_{Z+1,2}^{N}$ and ${\hat{L}}_{1,2}^{N}$ can be combined and then fed into the length-*Z* polar decoder to estimate ${\hat{u}}_{1,2}^{Z}$.

### 3.2. Information-Theoretic Finite-Length Code Performance

Given $y_{s}$, by using SIC, the received signal-to-interference-plus-noise ratio (SINR) at User 1 to detect the message of ${\bar{x}}_{1,1}^{Z}$ and User 2 to detect the message of ${\bar{x}}_{1,2}^{Z}$ in PC−SIC can be given by [26]

(16) $${\bar{\gamma }}_{1}=\frac{{\left|h_{1}\right|}^{2}P_{1}}{\sigma^{2}},$$

and

(17) $${\bar{\gamma }}_{2}=\frac{{\left|h_{2}\right|}^{2}P_{2}}{{\left|h_{2}\right|}^{2}P_{1}+\sigma^{2}}.$$

In PN−DNOMA, the BS transmits the superimposed message of the XORed message of two users and the message of the weak User 2. Thus, the XORed signal of the two transmitted messages is the message of User 1. From (14), we used the PNC mapping rule to obtain the information of User 1. By applying the cut−set theorem, the transmission rate of the XORed signal of the two transmitted messages of User 1 is given by

(18) $$C_{1}=\frac{1}{2}\log_{2}\left(1+\min\left(\frac{{\left|h_{1}\right|}^{2}P_{1}}{\sigma^{2}},\frac{{\left|h_{1}\right|}^{2}P_{2}}{\sigma^{2}}\right)\right),$$

where $C_{1}$ denotes the code rate of the XORed signal in User 1 and $\frac{{\left|h_{1}\right|}^{2}P_{1}}{\sigma^{2}}$ and $\frac{{\left|h_{1}\right|}^{2}P_{2}}{\sigma^{2}}$ denote the received SINR at User 1 to detect the message of $x_{1,1}^{Z}$ and $x_{1,2}^{Z}$, respectively [27,28]. Thus, the received SINR to detect the message of ${\bar{x}}_{1,1}^{Z}$ in PN−DNOMA is

(19) $${\hat{\gamma }}_{1}=\min\left(\frac{{\left|h_{1}\right|}^{2}P_{1}}{\sigma^{2}},\frac{{\left|h_{1}\right|}^{2}P_{2}}{\sigma^{2}}\right).$$

At User 2, from the polar decoding principle and Figure 4, both $L_{1,2}^{Z}$ and ${\hat{L}}_{1,2,xor}^{N}$ can obtain the source bits ${\hat{u}}_{1,2}^{Z}$ by utilizing the polar decoder with codelength *Z*, respectively. We assumed that the message ${\hat{u}}_{1,1}^{Z}$ from the BS is resolvable at User 2. Recall that we have two ways to decode the message $x_{1,2}^{Z}$ of User 2. Let $\frac{{\left|h_{2}\right|}^{2}P_{2}}{{\left|h_{2}\right|}^{2}P_{1}+\sigma^{2}}$ and $\frac{{\left|h_{2}\right|}^{2}P_{1}}{{\left|h_{2}\right|}^{2}P_{2}+\sigma^{2}}$ denote the received SINRs in these two decoding methods, respectively, which can be merged by maximal ratio combining (MRC) [29]. Thus, the overall SINR of User 2’s message in PN-DNOMA after MRC is

(20) $${\hat{\gamma }}_{2}=\frac{{\left|h_{2}\right|}^{2}P_{2}}{{\left|h_{2}\right|}^{2}P_{1}+\sigma^{2}}+\frac{{\left|h_{2}\right|}^{2}P_{1}}{{\left|h_{2}\right|}^{2}P_{2}+\sigma^{2}}.$$

As pointed out by [30], the decoding error probability at the receiver is non−negligible when the blocklength is finite. At user *s*, taking into account the impact of the non-zero error probabilities on decoding, we adopted the effective error probability $\epsilon_{s}$, given by

(21) $$\epsilon_{s}\approx Q\left(\ln2\sqrt{\frac{Z}{V_{s}}}\left(\log\left(1+\gamma_{s}\right)-R_{s}\right)\right),$$

where $\gamma_{s}\in \left\{{\bar{\gamma }}_{s},{\hat{\gamma }}_{s}\right\}$ denotes the received SINR over the PC−SIC and PN−DNOMA schemes, $R_{s}$ denotes the transmission rate, and $V_{s}$ is the channel dispersion parameter, given by

(22) $$V_{s}=1-{\left(1+\gamma_{s}\right)}^{-2},s\in \left\{1,2\right\}.$$

Furthermore, from the trade-off between the error probability and transmission rate, we used the effective throughput $T_{s}$ to evaluate the system with a finite blocklength. Mathematically, the effective throughput is defined by [31]

(23) $$T_{s}=\frac{Z}{r_{s}}R_{s}\left(1-\epsilon_{s}\right),s\in \left\{1,2\right\}.$$

In this system, our objective is to achieve user fairness and maximize the effective throughput $T_{t}$ by setting the power $P_{s}$, s∈{1,2}. Thus, the optimization for both PN−DNOMA and PC−SIC can be formulated as

(24) $$\begin{array}{c} \max_{\Delta }\;\;T_{t}=\left(T_{1}+T_{2}\right)/2\;\\ s.t.:\;\;P_{1}+P_{2}\leq P_{t}\;\\ \;\;\;\;\;\;\;\;\;\;R_{1}=R_{2}, \end{array}$$

where $\Delta =\{{\bar{\gamma }}_{1},{\bar{\gamma }}_{2},{\hat{\gamma }}_{1},{\hat{\gamma }}_{2}\}$ is the variable set that needs to be determined by PN−DNOMA and PC-SIC, respectively.

## 4. Simulation Results

**Figure 5:**
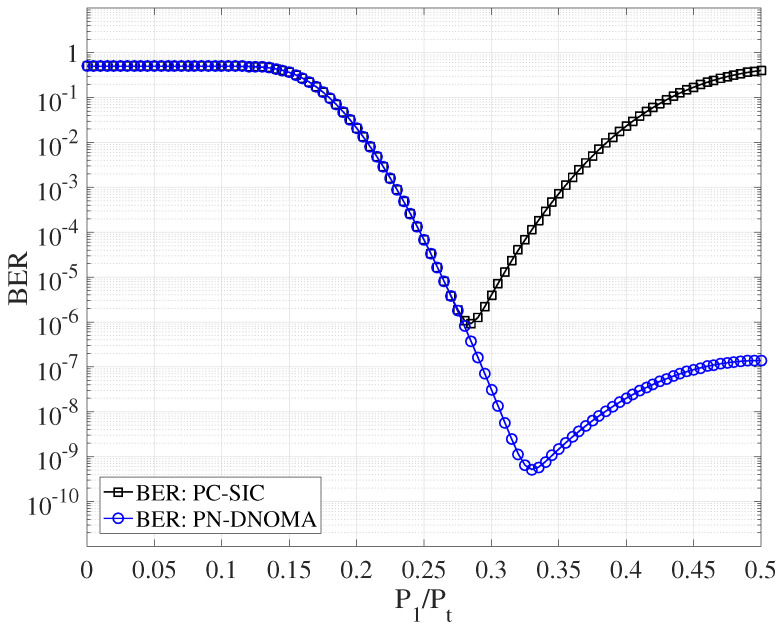
Error performance of two−user PN−DNOMA and PC−SIC with different power allocations at SNR = 18 dB.

This section simulates the error performances of the two-user downlink NOMA over quasi-static Rayleigh fading channels with $d_{1}=15$, $d_{2}=20$, and φ=0.5. Figure 5 shows the decoding error probability of PN−DNOMA and PC−SIC [12] with different power allocation ratios, at a code rate of 3/4 and SNR = 18 dB, respectively. Note that $P_{1}/P_{t}=0.28$ and $P_{1}/P_{t}=0.33$ are the optimal power allocation ratios for PN−DNOMA and PC−SIC, respectively. Moreover, the proposed PN−DNOMA shows a much lower error performance than the conventional PC−SIC when $P_{1}/P_{t}>0.28$.

**Figure 6:**
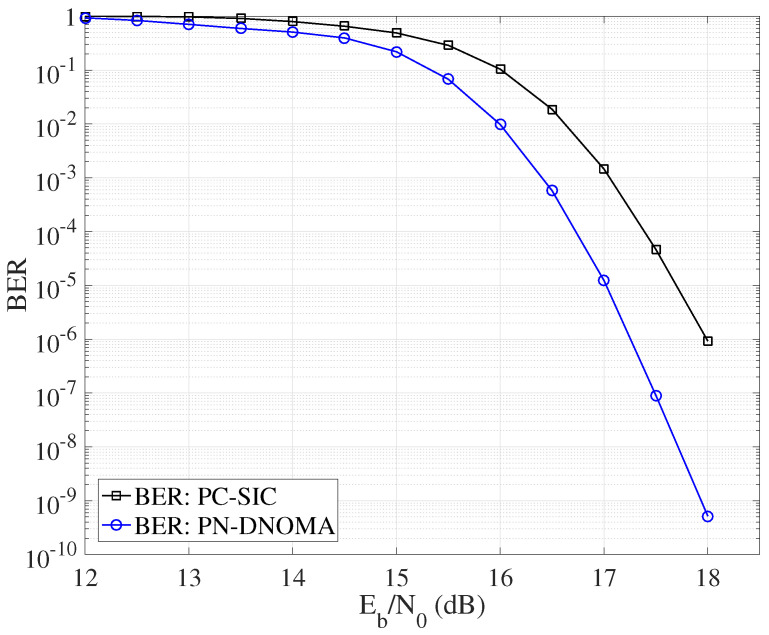
Error performance of PN−DNOMA and PC−SIC with the optimal power allocation, over two−user downlink NOMA.

Figure 6 further compares the decoding error probability of PN−DNOMA and PC−SIC with their optimal power allocation, respectively. The proposed PN−DNOMA can achieve a significant performance gain of 0.75 dB over PC−SIC, at a BER of $10^{-6}$ for Z=256.

**Figure 7:**
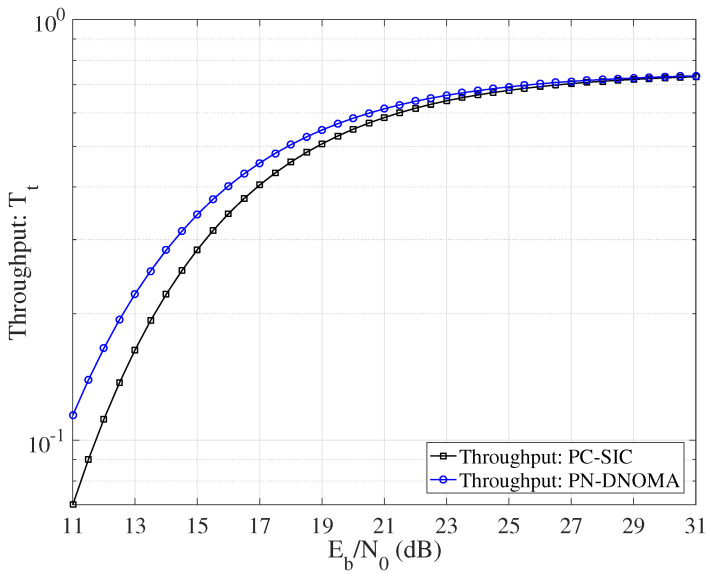
Effective throughput $T_{t}$ achieved by PN−DNOMA and PC−SIC, over two−user downlink NOMA.

Figure 7 shows the effective throughput $T_{t}$ achieved by PN−DNOMA and PC−SIC, respectively, for Z=256 and $R_{s}=3/4$. Note that all the benchmark coding schemes were optimized for the given channels. We can see that the proposed PN−DNOMA has a larger throughput $T_{t}$ than PC−SIC in all the SNR region.

**Figure 8:**
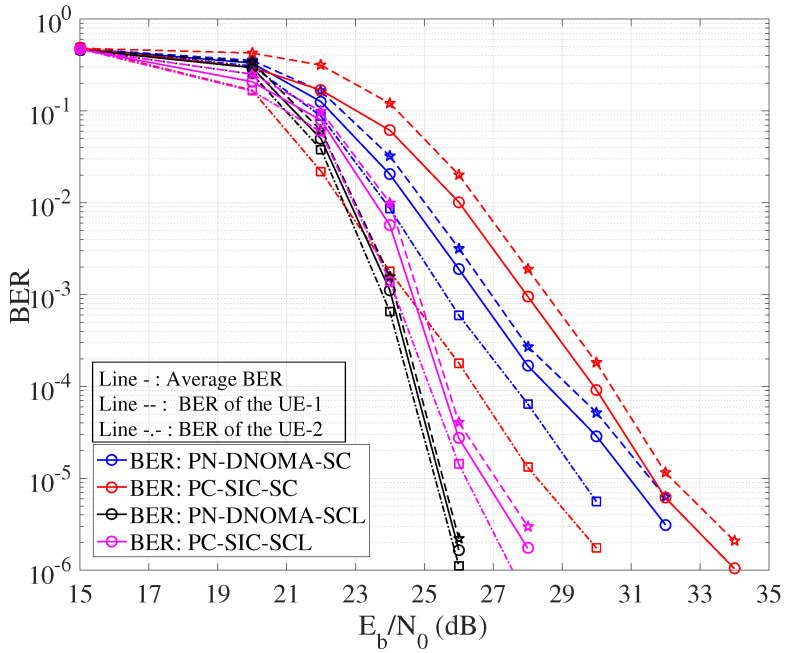
Error performance of PN−DNOMA and PC−SIC over two−user downlink NOMA.

Figure 8 shows the error performance of PN−DNOMA and PC−SIC. We adopted the SC decoder and CA−SCL decoder at the user receivers, respectively. In particular, CA−SCL uses list size L=4 and CRC-8 with the generator polynomial $g\left(x\right)=x^{8}+x^{2}+x+1$. In PC−SIC, a joint successive cancellation (JSC) decoding is used [12]. The code rates of the two users are $\left(R_{1},R_{2}\right)=\left(0.75,0.75\right)$ and Z=256 for both PN−DNOMA and PC−SIC. The proposed PC−DNOMA can achieve a significant performance gain of 0.7 dB and 0.45 dB over PC−SIC for both SC and CA−SCL decoders, at a BER of $10^{-5}$, as shown in Table 2. Moreover, the performance gap of User 1 and User 2 in PN−DNOMA is smaller than that of PC−SIC, which demonstrates its enhanced user fairness.

With respect to the decoding complexity, the proposed PN−DNOMA consists of the PNC mapping operation and polar decoding with a length−*Z* decoder at User 1. In the receiver for User 2, we constructed a length−*N* polar decoder. Thus, the decoding complexity of PN−DNOMA with the SC and CA−SCL decoders is NlogN+2Nlog2N and LNlogN+2LNlog2N, respectively, while in PC−SIC, a JSC decoding is used for User 1 and User 2, directly recovering the message of User 2 from the superimposed messages. Thus, the decoding complexity of PC−SIC with the SC and CA−SCL decoders is 4NlogN and 4LNlogN, respectively. This means that the proposed PN−DNOMA can improve the decoding performance without increasing the complexity.

## 5. Conclusions

In this paper, we proposed a PN−DNOMA for two−user downlink NOMA systems, where the BS broadcasts the superimposed message of the XORed message of two users and the message of the weak User 2. The main finding of this paper is that, by exploiting the polarization effect and PNC principle, we can use polar decoding to directly recover the message of User 1, while for User 2, a long−length polar code from the superimposed message can be constructed. In this way, the message of User 2 can be recovered by this polar code as a multiuser decoding. The channel polarization effect of the polar decoding in PN−DNOMA is greatly improved. The simulation results showed that the proposed PC−DNOMA can achieve significant performance gains of 0.7 dB and 0.45 dB over the conventional PC−SIC for the SC and CA−SCL decoders, at a BER of $10^{-5}$, respectively, and the user fairness is also enhanced over two−user NOMA systems. Furthermore, as a potential direction, it is worthwhile to mention that the proposed scheme can be extended to more generalized *M*−user NOMA, M>2, to enhance the system performance in the future.

## Figures and Tables

**Table 1:** Mapping rules for code bits and received signals.

*j*	$x_{1}$	$x_{2}$	$x_{o}$	$h_{s}\sqrt{p_{1}}r_{1}$	$h_{s}\sqrt{p_{2}}r_{2}$	*S*
0	0	0	0	$h_{s}\sqrt{p_{1}}$	$h_{s}\sqrt{p_{2}}$	$h_{s}\left(\sqrt{P_{1}}+\sqrt{P_{2}}\right)$
1	1	0	1	$-h_{s}\sqrt{p_{1}}$	$h_{s}\sqrt{p_{2}}$	$h_{s}\left(-\sqrt{P_{1}}+\sqrt{P_{2}}\right)$
2	0	1	1	$h_{s}\sqrt{p_{1}}$	$-h_{s}\sqrt{p_{2}}$	$h_{s}\left(\sqrt{P_{1}}-\sqrt{P_{2}}\right)$
3	1	1	0	$-h_{s}\sqrt{p_{1}}$	$-h_{s}\sqrt{p_{2}}$	$-h_{s}\left(\sqrt{P_{1}}+\sqrt{P_{2}}\right)$

**Table 2:** Error performance and decoding complexity of PN−DNOMA and PC−SIC over two−user downlink NOMA.

	Decoder			
	SC Decoder		CA−SCL Decoder	
	PC−SIC	PN−DNOMA	PC−SIC	PN−DNOMA
BER: $1.0\times 10^{-4}$	30.1 (dB)	28.3 (dB)	25.6 (dB)	24.5 (dB)
BER: $1.0\times 10^{-5}$	31.8 (dB)	30.9 (dB)	26.7 (dB)	25.6 (dB)
Decoding Complexity	4NlogN	NlogN+2Nlog2N	4LNlogN	LNlogN+2LNlog2N

## Data Availability

The data are contained within the article.

## Abbreviations

The following abbreviations are used in this manuscript:

PNC	Physical network coding
PN-DNOMA	A joint PNC for two−user downlink non−orthogonal multiple access
NOMA	Non−orthogonal multiple access
SIC	Successive interference cancellation
PA	Power allocation
BS	Base station
PC-SIC	A joint polar decoding and SIC decoding strategy
NCMA	Network-coded multiple access
SC	Successive cancellation
CS-SCL	Cyclic−redundancy−check−aided SC−list
CSIT	Channel state information at the transmitter
B-DMC	Binary−input discrete memoryless channel
SINR	Signal−to−interference−plus−noise ratio
JSC	Joint successive cancellation
QAM	Quadrature amplitude modulation
MF	Matched-filter
